# Naturally Colored ZrO_2_-Based Hybrids Loaded with Olive Leaf Extract: Physicochemical, Thermal and Release Properties

**DOI:** 10.3390/molecules31173020

**Published:** 2026-08-28

**Authors:** Marika Fiorentino, Maria Vittoria Mollo, Antonio D’Angelo, Daniele Naviglio, Ignazio Blanco, Michelina Catauro

**Affiliations:** 1 Department of Engineering, University of Campania “Luigi Vanvitelli”, Via Roma 29, 81031 Aversa, CE, Italy; marika.fiorentino@unicampania.it (M.F.); mariavittoria.mollo@unicampania.it (M.V.M.); 2 Department of Industrial Engineering, University of Salerno, Via Giovanni Paolo II, 84084 Fisciano, SA, Italy; andangelo@unisa.it; 3 Department of Chemical Sciences, University of Naples “Federico II”, Via Cinthia, 26, 80126 Napoli, NA, Italy; daniele.naviglio@unina.it; 4 Department of Civil-Industrial Engineering and Architecture, University of Catania, Via Santa Sofia 64, 95125 Catania, CT, Italy; iblanco@unict.it

**Keywords:** zirconia, hybrids, olive leaf, sol–gel synthesis, controlled release, colorimetric analysis, TG/DSC, antibacterial activity

## Abstract

Olive leaf extract (OLE) contains polyphenolic compounds with reported antioxidant and antimicrobial activities. In this study, OLE obtained by Naviglio extraction was incorporated into zirconia-based sol–gel hybrids at theoretical contents of 8, 25, 33, and 50 wt%. Colorimetric analysis showed that OLE acted as a natural coloring agent, producing composition-dependent changes from yellow to orange–brown tones. FT-IR spectra retained the characteristic bands of the zirconia-based matrix together with OLE-related contributions, while shifts in the O–H and Zr–O regions indicated changes in the local chemical environment after incorporation. Thermal analysis showed a progressive increase in the onset temperature of the main degradation stage, from 296.2 °C for bare ZrO_2_ to 320.4 °C for ZrO_2_/OLE50%, suggesting a stabilizing effect of OLE within the hybrid matrix. Release experiments in 95% (*v*/*v*) ethanol showed a clear dependence on OLE content. ZrO_2_/OLE8% released almost completely within 5–6 h, whereas ZrO_2_/OLE25% showed an initial burst followed by slower release, reaching 75–80% after 24 h. ZrO_2_/OLE33% and ZrO_2_/OLE50% showed slower release without a clear burst phase and released less than 30%. Agar-diffusion tests showed no enhancement of antibacterial activity against *Escherichia coli* or *Enterococcus faecalis*. These preliminary findings provide a basis for further investigation into the valorization of olive leaf extract as a natural component for the development of colored functional hybrid materials.

## 1. Introduction

Active food-packaging systems are designed to limit oxidative degradation and microbial growth, extending food shelf life while reducing the use of synthetic preservatives [1]. Plant-derived compounds recovered from agro-industrial by-products can contribute to this function because of their antioxidant and antimicrobial properties [2]. Olive leaves (*Olea europaea* L.), an abundant by-product of the olive oil industry [3], contain several phenolic compounds, including oleuropein, hydroxytyrosol, tyrosol, verbascoside, and flavonoids [4,5]. These compounds make olive leaf extract (OLE) a promising natural ingredient for food-preservation applications [6]. The recovery of OLE also supports the valorization of agricultural residues within a circular economy approach. However, the direct incorporation of OLE into packaging materials may be limited by the susceptibility of its phenolic compounds to oxidation, light, and thermal degradation [7,8]. Thus, the possibility of encapsulating or immobilize within a suitable matrix, therefore, improves the protection of the extract and modulates its release over time [9].

In this context, the sol–gel technique enables the entrapment of thermolabile and unstable bioactive compounds to form organic–inorganic hybrids under mild conditions, allowing thermolabile compounds to be incorporated during network formation [10,11]. The composition and structure of the inorganic network, together with the interactions established with the organic phase, influence molecular mobility and therefore the retention and release of the incorporated compounds [12].

Zirconia (ZrO_2_) provides a chemically stable inorganic phase with good mechanical resistance and biocompatibility [13]. When synthesized by the sol–gel route, its network can be formed under mild conditions and modified through precursor composition and synthesis parameters. These factors influence the distribution, retention, and mobility of the incorporated organic phase [14,15]. The composition of a plant extract depends strongly on the extraction conditions. Conventional solvent-based methods often require long processing times, large solvent volumes, or prolonged heating. Ultrasound- and microwave-assisted extraction can reduce extraction time, although local heating may affect thermolabile compounds [16]. In this context, the Naviglio extraction is based on repeated pressure and decompression cycles that promote solvent penetration and solute transfer from the plant matrix. Since the extraction is performed at or near room temperature, thermal exposure is limited [17]. This method has been applied to the recovery of phenolic compounds from olive leaves and other plant materials. In this study, OLE obtained by Naviglio extraction was incorporated into zirconia-based hybrids at different loadings of 8, 25, 33, and 50 wt%. The materials were analyzed by colorimetry, FTIR spectroscopy, thermal analysis, release tests in 95% ethanol, and agar-diffusion assays against *Escherichia coli* and *Enterococcus faecalis*. Particular attention was given to the ability of the natural extract to modify the colorimetric properties of the zirconia-based matrix. The study examined the effect of OLE loading on the color, physicochemical characteristics, thermal behavior, release profile, and antibacterial response of the hybrids.

## 2. Results and Discussion

### 2.1. Sol–Gel Synthesis and Visual Appearance of Zirconia Hybrids

**Figure 1:**
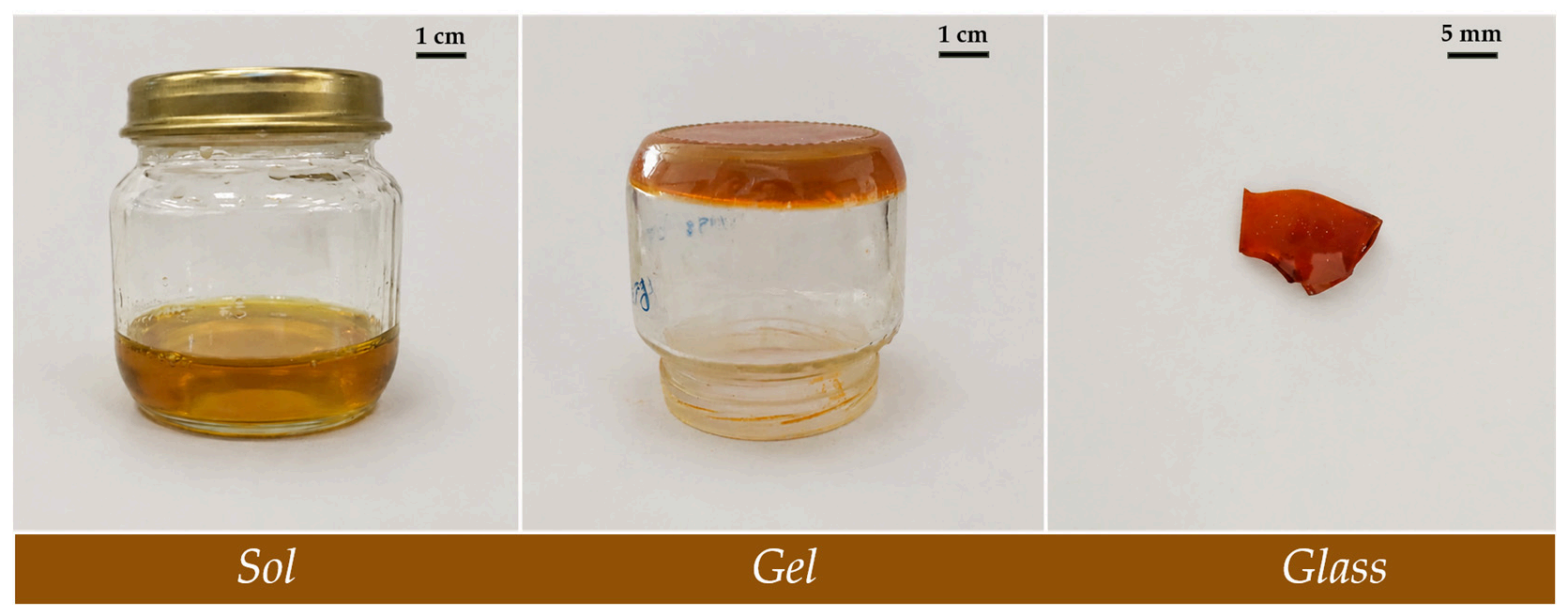
Representative images of the ZrO_2_/OLE hybrids in the sol, gel, and glass phases. ZrO_2_/OLE33% is shown as a representative formulation of the entire hybrid series.

**Figure 2:**
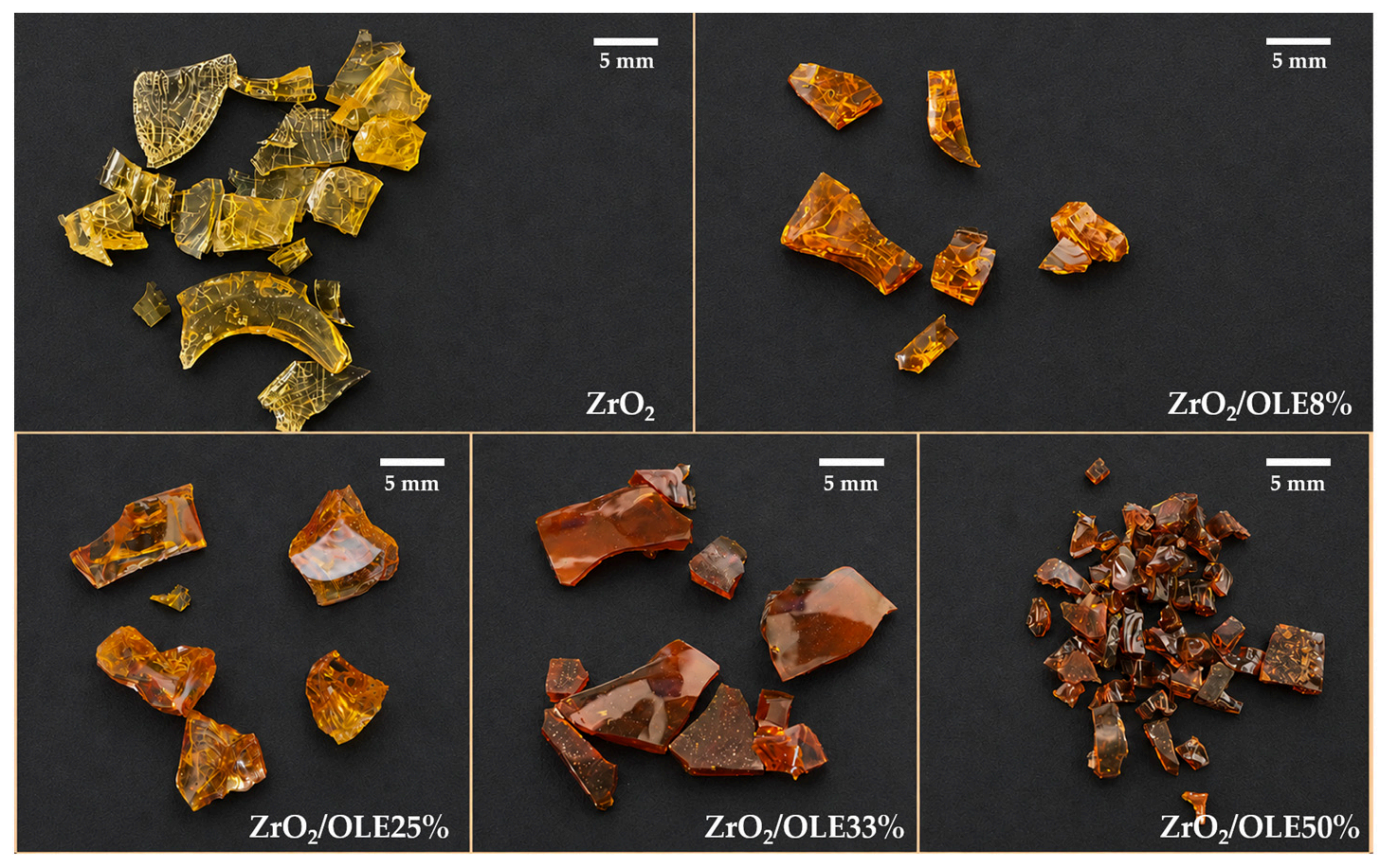
Representative images of ZrO_2_ and ZrO_2_/OLE hybrids.

Zirconia-based hybrids were synthesized via the sol–gel method to incorporate olive leaf extract (OLE). The green-colored OLE was first dissolved in ethanol and subsequently added to the inorganic phase, consisting of zirconium propoxide, acetylacetone, and ethanol. The zirconia precursor solution exhibited a yellow color, which was retained in the dried bare ZrO_2_, whereas the addition of OLE induced a yellow–green coloration with a slight olive hue (Figure 1). During gelation, the color progressively shifted toward orange and was retained after vacuum drying (Figure 2). The final coloration varied with OLE loading, indicating that the extract contributed to the color of the zirconia-based matrix. These differences were subsequently quantified by CIELAB analysis.

### 2.2. Colorimetric Analysis

**Figure 3:**
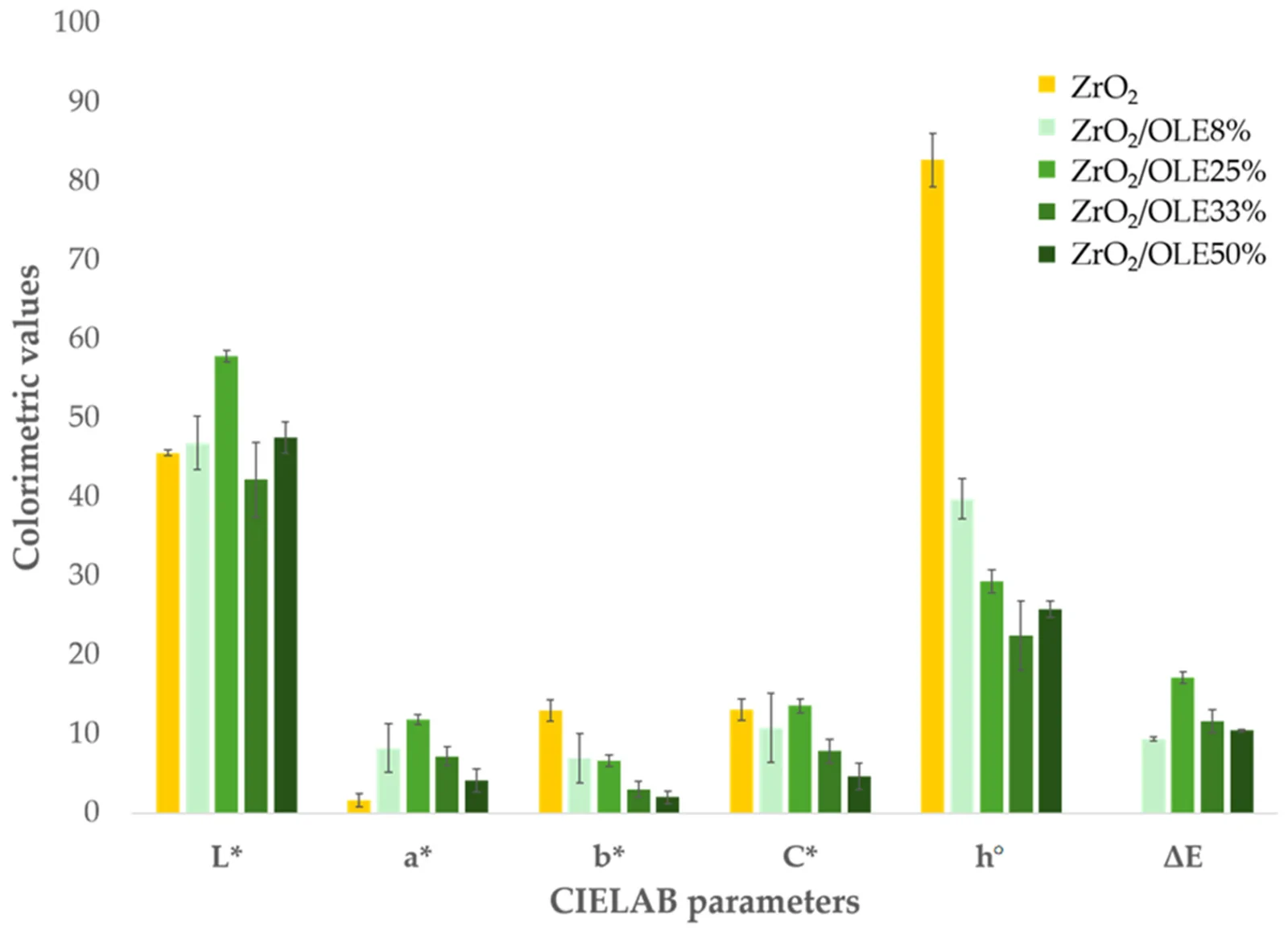
Colorimetric analysis of ZrO_2_/OLE hybrids. The CIELAB parameters include lightness (L*), the green–red coordinate (a*), the blue–yellow coordinate (b*), chroma or color saturation (C*), hue angle (h°), and total color difference (ΔE) calculated with respect to pure ZrO_2_. Data are reported as mean ± standard deviation (SD) from three independent measurements performed on three different specimens (*n* = 3).

Once the dried materials were obtained, colorimetric analysis was performed using the CIELAB system to obtain an objective and standardized description of the color of the hybrids. Figure 3 reports the variations in the L*, a*, b*, C*, and h° parameters. The L* parameter describes material lightness. Pure ZrO_2_ and ZrO_2_/OLE8% show similar values, whereas ZrO_2_/OLE25% exhibits the highest L* value, in agreement with its brighter and more vivid orange appearance. At higher OLE contents, L* decreases, indicating that ZrO_2_/OLE33% and ZrO_2_/OLE50% reflect less light and therefore appear darker, likely because of the higher amount of colored OLE-derived compounds incorporated into the glass matrix. All a* values are positive, indicating that hybrids lie on the red side of the green–red axis. The a* value increases up to 25% OLE, consistent with the development of a stronger orange–red component, and then decreases at higher OLE concentrations. The b* coordinate remains positive for all hybrids, confirming the persistence of a yellow component, but progressively decreases with increasing OLE content. The chroma C* reaches its highest value for ZrO_2_/OLE25%, reflecting its more vivid and saturated orange color, whereas lower values are observed for ZrO_2_/OLE33% and ZrO_2_/OLE50%, which appear darker and less saturated. Finally, the progressive decrease in hue angle h° confirms the color shift from yellow in pure ZrO_2_ toward orange and orange–red tones in the OLE-containing hybrids. The ΔE parameter represents the overall color difference between each hybrid glass and the reference sample, in this case pure ZrO_2_. Since ΔE values greater than five are generally considered clearly perceptible to the human eye, the measured values confirm the color differences observed qualitatively [18,19]. The highest ΔE value was obtained for ZrO_2_/OLE25%, mainly due to its higher lightness and a* coordinate compared with pure ZrO_2_.

### 2.3. FT-IR Analysis

**Figure 4:**
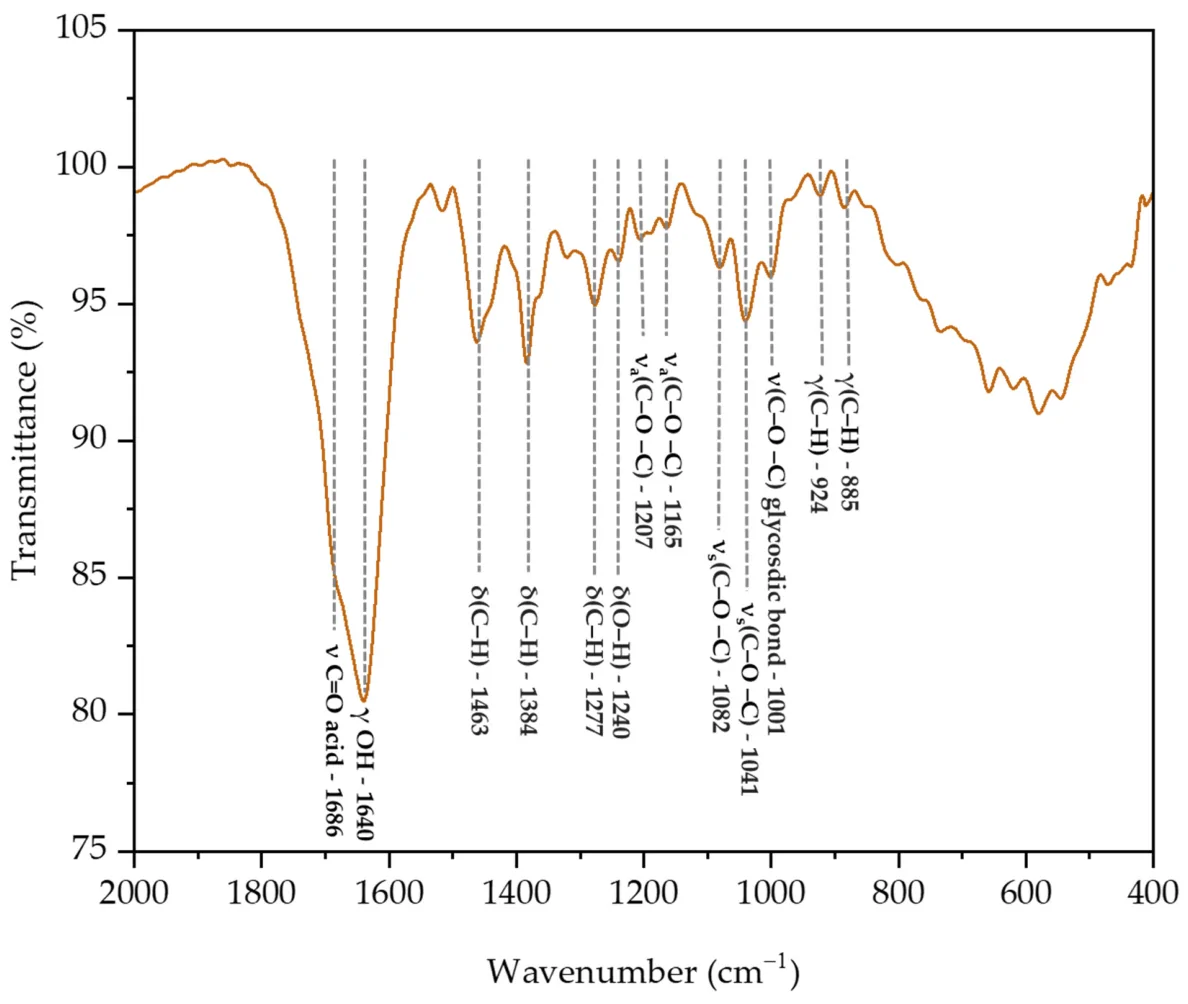
OLE FTIR spectrum in the 2000–400 cm^−1^ range.

The FT-IR spectrum of OLE was recorded and is shown in Figure 4. In accordance with the literature, the OLE is mainly composed of bioactive polyphenolic compounds characteristic of *Olea europaea* leaves. The predominant compound is oleuropein, belonging to the class of oleuropeosides, which represents the major phenolic constituent of olive leaves. In addition, the extract contains flavan-3-ols, such as catechin, together with simple phenolic compounds including tyrosol, hydroxytyrosol, vanillin, vanillic acid, and caffeic acid. The FT-IR spectrum of OLE is shown in the 2000–400 cm^−1^ range to provide a more detailed analysis of the vibrational bands in the fingerprint region, particularly below 1500 cm^−1^. In the fingerprint region, the signal at 1686 is associated with C=O stretching of conjugated carbonyl/carboxylic groups, while the band at 1640 cm^−1^ is assigned to O–H bending vibrations. The bands at 1463 and 1384 cm^−1^ are attributed to C–H bending vibrations, while the peaks at 1277, 1240, 1207, and 1165 cm^−1^ are related to C–O stretching and O–H bending modes of phenolic alcohols, phenolic acids, and flavonoid-related compounds [20]. Additional signals at 1082 and 1041 cm^−1^ are assigned to C–O vibrations, whereas the weak band at 1001 cm^−1^ is related to C–O–C vibrations of glycosidic bonds. The low intensity of this latter signal may suggest a limited contribution of glycosidic or polysaccharidic structures, indicating that ethanol extraction assisted by the Naviglio extractor mainly favored the selective recovery of phenolic compounds. Finally, the bands at 924, 885, and 833 cm^−1^, together with those in the lower wavenumber region, can be associated with out-of-plane C–H bending and skeletal deformation modes of substituted aromatic rings [21]. Overall, these assignments should be regarded as tentative, since FT-IR spectroscopy provides preliminary information on the main functional groups present in the extract, allowing an initial assessment of its chemical profile. However, unambiguous identification and quantification of individual phenolic compounds require dedicated compositional analyses, such as chromatographic methods.

**Figure 5:**
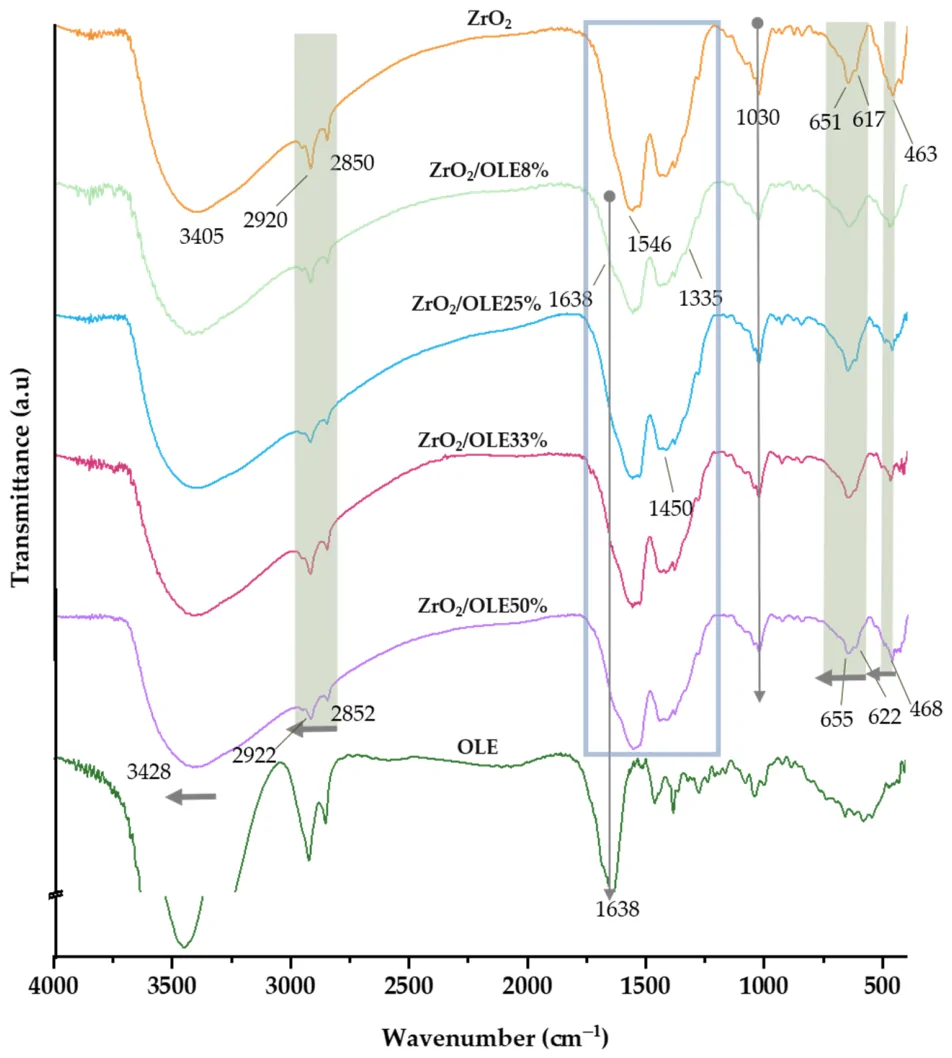
FTIR spectra of ZrO_2_/OLE hybrids. ZrO_2_ and OLE are reported for comparison. The blue outlined rectangle indicates the AcAc-related spectral region, the green rectangles highlight the observed blue shifts, and the grey arrow indicates peaks that do not show any shift.

The FT-IR spectra of ZrO_2_/OLE hybrids are reported in Figure 5, together with those of pure OLE and ZrO_2_ for comparison. The spectra were examined to assess the incorporation of the extract within the inorganic zirconia matrix and to investigate the formation of the hybrid system. All ZrO_2_/OLE hybrids retained the main absorption bands characteristic of ZrO_2_, confirming the preservation of the inorganic network. In particular, the broad O–H stretching band, centered at approximately 3405 cm^−1^ in the ZrO_2_ reference, is attributed to surface Zr–OH groups involved in hydrogen bonding and to adsorbed water molecules [22]. After OLE incorporation, it shifted to about 3428 cm^−1^ in ZrO_2_/OLE50%, suggesting a partial disruption and reorganization of the hydrogen-bonding network involving Zr–OH groups and adsorbed water following OLE incorporation.

The bands observed at approximately 2920 and 2850 cm^−1^ in bare ZrO_2_, assigned to the asymmetric and symmetric aliphatic C–H stretching vibrations of residual acetylacetone, respectively, are partially overlapped in the hybrid spectra with C–H stretching contributions from the organic compounds present in OLE. In the hybrid materials, these bands were observed at approximately 2922 and 2852 cm^−1^, respectively. Therefore, their spectral evolution likely reflects the superposition of acetylacetone-derived and OLE-derived organic compounds. On the other hand, the bands observed at approximately 651 and 617 cm^−1^, assigned to Zr–OH and Zr–O–Zr vibrations, respectively, progressively shifted towards higher wavenumbers with increasing OLE content, reaching 655 and 622 cm^−1^, respectively, for ZrO_2_/OLE50% [23]. Overall, these progressive shifts indicate that OLE encapsulation modifies the local chemical and coordination environment of both the acetylacetone-derived groups and the zirconia network. Finally, the band observed at 463 cm^−1^ was assigned to O–Zr–O bending vibrations [24] (in bare ZrO_2_), confirming the formation of Zr–O–Zr linkages within the amorphous zirconia-based matrix that shifted toward 468 cm^−1^ in ZrO_2_/OLE50%.

The interactions established between OLE functional groups and the ZrO_2_ matrix result in slightly more rigid vibrational environments [25]. Moreover, the contribution associated with the C=O stretching vibrations of OLE polyphenolic compounds appeared in the hybrid spectra as a shoulder (1638 cm^−1^) [26] on the intense acetylacetone-related band centered at approximately 1546 cm^−1^. Although partially overlapped, the emergence of this feature supports the incorporation of OLE organic compounds into the ZrO_2_ matrix.

### 2.4. Thermal Analysis

**Figure 6:**
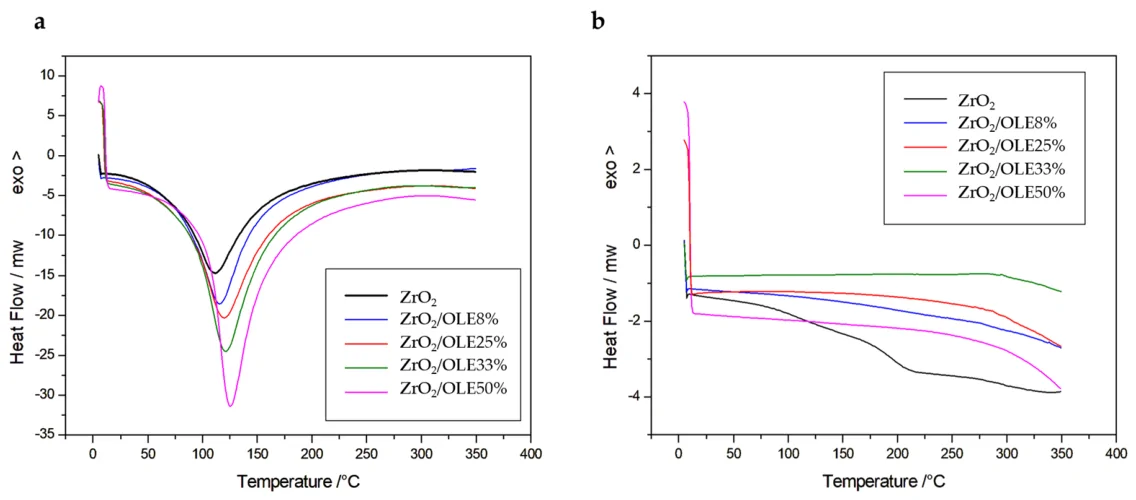
(**a**) DSC heating curves at 10 °C·min^−1^, in the static air atmosphere, for the ZrO_2_ and ZrO_2_/OLE hybrids. (**b**) DSC curves recorded for the ZrO_2_ and ZrO_2_/OLE hybrids, after cooling from the first scan under the same conditions.

Calorimetric analyses of powder ZrO_2_ and ZrO_2_/OLE hybrids at different percentages of OLE are reported in Figure 6a, showing a marked endothermic peak due to the water loss just above 100 °C. The second DSC scan, performed on all the samples after cooling down to room temperature (Figure 6b), shows the disappearance of the endothermic peak, thus confirming the water loss [27].

**Figure 7:**
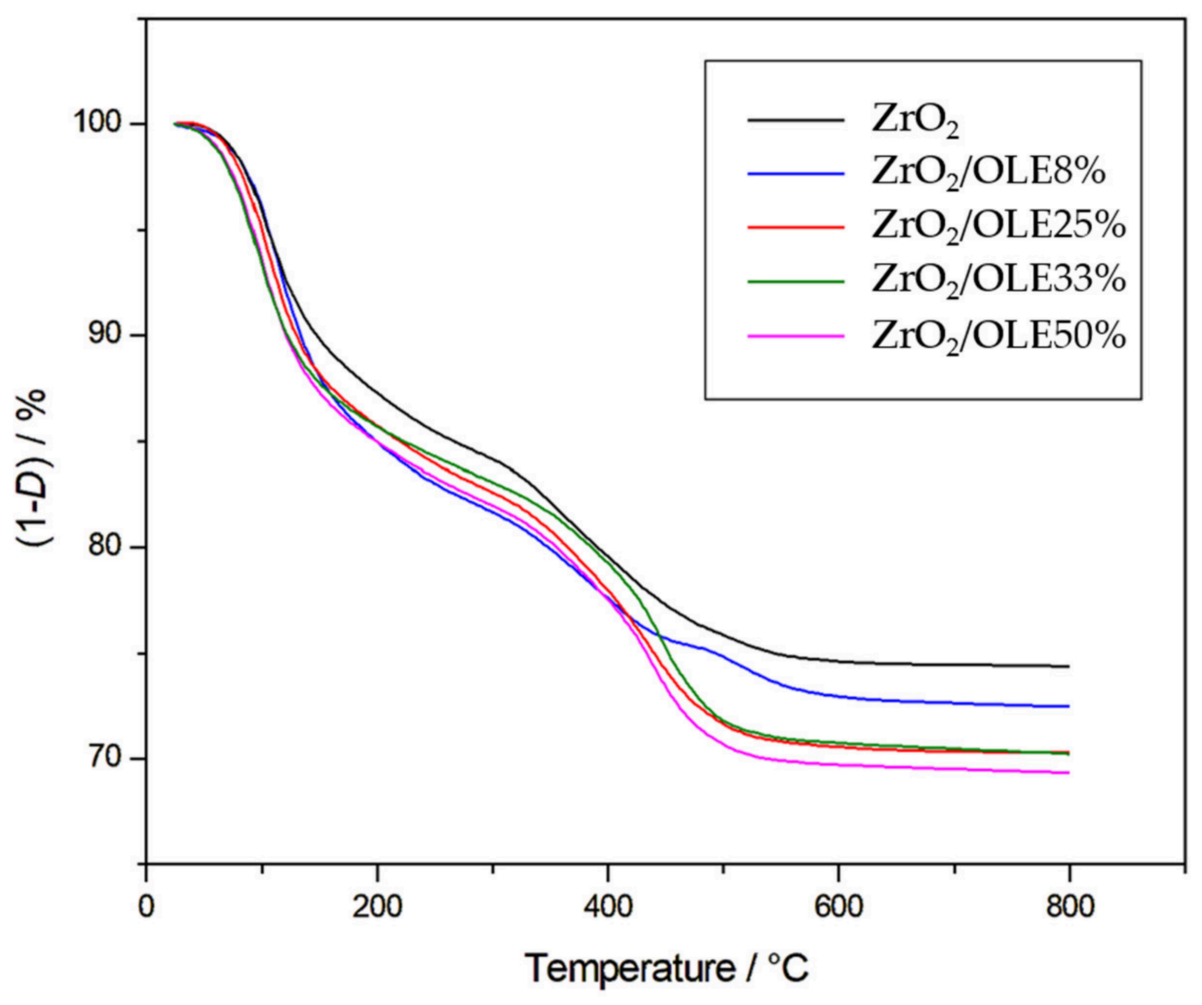
TG degradation curves of ZrO_2_/OLE hybrid and ZrO_2_ at 10 °C min^−1^ under nitrogen flow.

As reported in Figure 6a, the endothermic peak is slightly shifted towards a higher temperature by increasing the OLE percentage in the hybrid, and the enthalpy of the peak increases following the same trend. Peak temperatures of the DSC phenomena are reported in Table 1, together with TG thermal parameters. ZrO_2_ and ZrO_2_/OLE hybrids were then degraded in the thermobalance up to 800 °C in an inert environment, and the TG degradation curves are reported in Figure 7.

For all the hybrids and ZrO_2_, a first degradation stage, from about 100 °C to 150 °C, was recorded, and it was attributed to water loss, in agreement with the DSC run (Figure 6a). Water loss affected the temperature at 5% mass loss (*T*_5%_), generally considered as a measure of the resistance to thermal degradation. Thus, the initial decomposition temperature (*T*_i_), determined as the intersection between the starting mass line and the maximum gradient tangent to the TG curve, was considered as a measure of the thermal resistance in this case. As we can see from Table 1, it is likely that the moisture of the hybrids increases as a function of the OLE percentage, thus lowering the *T*_5%_ values. ZrO_2_/OLE hybrids and ZrO_2_ presented a sharp degradation stage, whose beginning is shifted toward higher temperatures as a function of OLE content increase (Table 1)**,** thus showing a stabilizing effect of the interactions within the hybrid matrix. Yuan et al. reported that a polyphenol-rich olive leaf extract began to decompose at approximately 190 °C, with DTG maxima at 216.5 and 378.5 °C. Although a direct comparison is limited by the different extraction and experimental conditions, the main degradation stage occurred above 300 °C in the ZrO_2_/OLE hybrids and shifted progressively toward higher temperatures as the OLE content increased. This trend suggests that the interactions established between OLE and the zirconia matrix contributed to delaying the main thermal degradation process [28]. By contrast, the percentage of the solid residue obtained at 800 °C decreased with the increase in the OLE content, as we expected.

### 2.5. In Vitro Release

**Figure 8:**
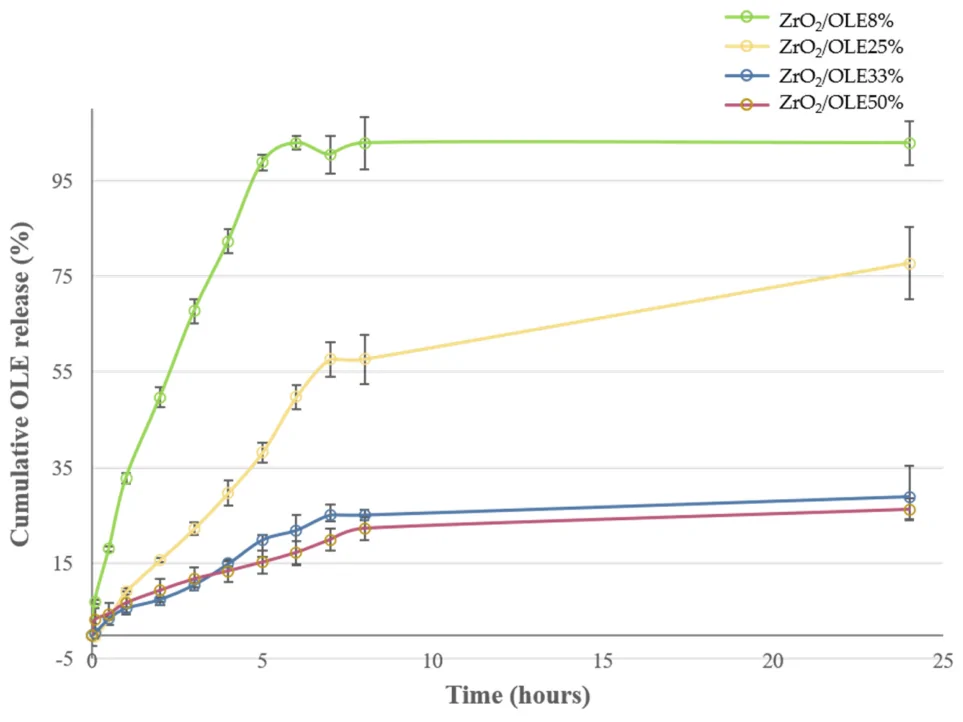
Cumulative release in % of ZrO_2_/OLE hybrids. Error bars represent the standard deviation of three independent measurements (*n* = 3).

The release of OLE from the zirconia-based hybrids was evaluated in 95% (*v*/*v*) ethanol over 24 h. The experiment was designed as a preliminary study to investigate the release behavior of the incorporated extract under conditions relevant to contact with lipophilic foods, such as edible oils. Concentrated ethanol was selected as the release medium because it is used as a substitute for food simulant D2 in migration studies involving fatty-food contact materials [29]. As shown in Figure 8, the release behavior strongly depended on the amount of OLE added during synthesis. ZrO_2_/OLE8% showed a rapid and nearly complete release, reaching approximately 100% of the theoretical OLE content within 5–6 h, followed by a plateau. This behavior indicates that most of the extract present in this formulation was readily accessible to the ethanolic medium and rapidly released from the hybrid matrix [30]. ZrO_2_/OLE25% showed a different release pattern, characterized by an initial burst release followed by a slower and more controlled release phase. Approximately 58% of the theoretical OLE content was released within the first 7 h, after which the release proceeded more gradually, reaching approximately 75–80% after 24 h. The initial burst may be associated with an OLE fraction located at or near the material surface or in readily accessible regions of the matrix, whereas the subsequent slower release suggests that a second fraction was more strongly retained within the zirconia-based network. Although burst release is often considered undesirable in controlled-release systems, rapid initial delivery may be useful in food-contact applications when early availability of the active compound is required [31]. In contrast, ZrO_2_/OLE33% and ZrO_2_/OLE50% do not show an evident burst-release phase. Both formulations displayed a slower release over time, progressively approaching a plateau while releasing less than 30% of the theoretical amount of OLE added during synthesis within 24 h. This behavior indicates that, at higher OLE contents, a larger fraction of the extract remained retained within the hybrid matrix. Stronger interactions with the inorganic network or the formation of less accessible OLE-rich regions may contribute to this retention. The results show that OLE loading markedly affected both the rate and extent of release. ZrO_2_/OLE25% was the only formulation showing a clear initial burst followed by a prolonged slower release, whereas higher OLE contents resulted in slower and incomplete release within the investigated period.

### 2.6. Antibacterial Evaluation

**Figure 9:**
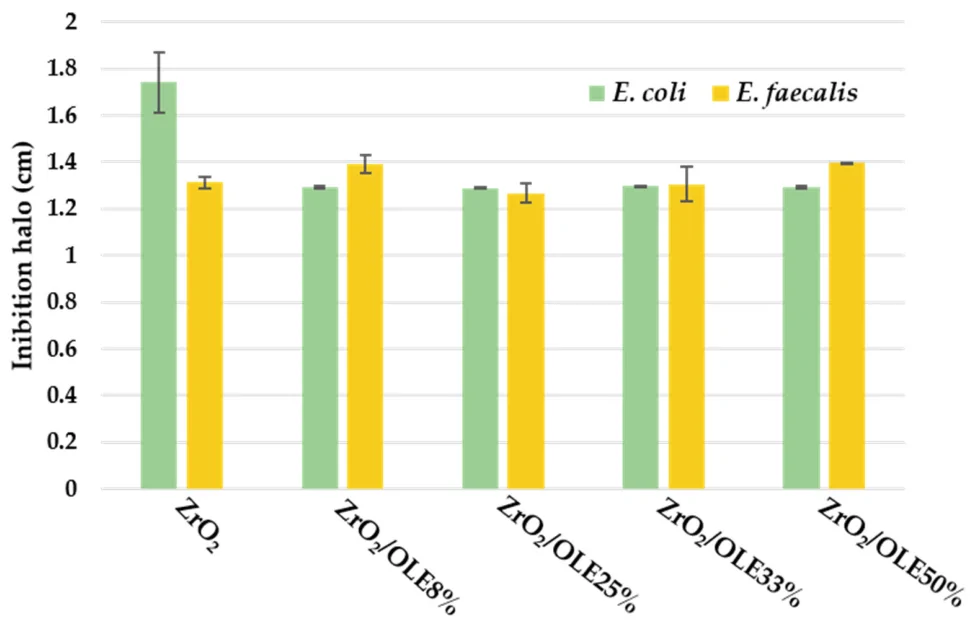
Inhibition halos of ZrO_2_ and ZrO_2_/OLE hybrids against *Escherichia coli* and *Enterococcus faecalis* bacterial strains.

The antibacterial response of bare ZrO_2_ and ZrO_2_/OLE hybrids was evaluated against *Escherichia coli* and *Enterococcus faecalis* using an agar-diffusion assay (Figure 9). Against *E. coli*, bare ZrO_2_ produced an inhibition zone of approximately 1.7 cm, slightly larger than the 1.3 cm diameter of the pressed pellet prepared from the sample for antibacterial testing. This result indicates a limited inhibitory effect extending beyond the pellet perimeter, in agreement with antibacterial activity previously reported for zirconia-based materials [27,32]. After OLE incorporation, no additional inhibition zone was observed in the surrounding agar, indicating that, under the experimental conditions used, the ZrO_2_/OLE hybrids did not produce measurable diffusion-mediated inhibition against *E. coli*. A similar behavior was observed against *E. faecalis*; in this case, however, bare ZrO_2_ as well as all ZrO_2_/OLE hybrids showed an inhibition halo of approximately 1.3 cm, corresponding to the diameter of the pressed sample pellet. Relevant antibacterial activity of olive leaf extracts against both *E. coli* and *E. faecalis* has been reported in the literature, although the extent of inhibition depends on extract composition, extraction solvent, concentration, and bacterial strain [32,33,34]. In the present study, OLE entrapped within the zirconia-based matrix was not able to generate an inhibition zone beyond the pellet perimeter, regardless of the OLE percentage incorporated during synthesis. This result suggests that OLE immobilization within the inorganic matrix limited the availability of antibacterial compounds for diffusion into the agar, or that the amount released under these conditions remained below the concentration required to inhibit bacterial growth. Accordingly, the hybrids did not exert a detectable antibacterial effect against either strain beyond the area directly occupied by the sample.

## 3. Materials and Methods

### 3.1. Naviglio Extraction

Olive leaf extract (OLE) was obtained using Naviglio Extraction technology, an innovative solid–liquid extraction technique based on the generation of a negative pressure gradient between the interior of the plant matrix and the surrounding extraction solvent [35]. This pressure difference promotes the rapid migration of compounds that are not chemically bound to the solid matrix, allowing efficient extraction under mild operating conditions. Unlike conventional extraction methods, which primarily rely on diffusion and osmosis and frequently require elevated temperatures to increase extraction yields, the Naviglio Extraction process employs alternating static and dynamic pressure cycles to enhance mass transfer. Consequently, thermolabile bioactive compounds are efficiently recovered while minimizing thermal degradation, extraction time, solvent consumption, and energy requirements [36]. These features make the technology a sustainable and efficient alternative to conventional solid–liquid extraction techniques. Fresh olive leaves were thoroughly washed with distilled water, air-dried at room temperature, and coarsely ground to increase the contact surface with the extraction solvent. The plant material was then mixed with ethanol and processed using a 2 L Naviglio Extractor^®^ (Atlas Filtri Engineering, Limena, Italy). The extraction protocol consisted of alternating static and dynamic phases of 2 min each, repeated for 30 consecutive cycles, corresponding to a total extraction time of 2 h. At the end of the extraction process, the ethanolic extract enriched in polyphenolic compounds was separated from the exhausted plant material and filtered through qualitative filter paper to remove residual solid particles. The resulting olive leaf extract was stored at 4 °C until further use in the preparation of the zirconia-based hybrid materials.

### 3.2. Synthesis Sol–Gel of ZrO_2_/OLE Hybrids

**Figure 10:**
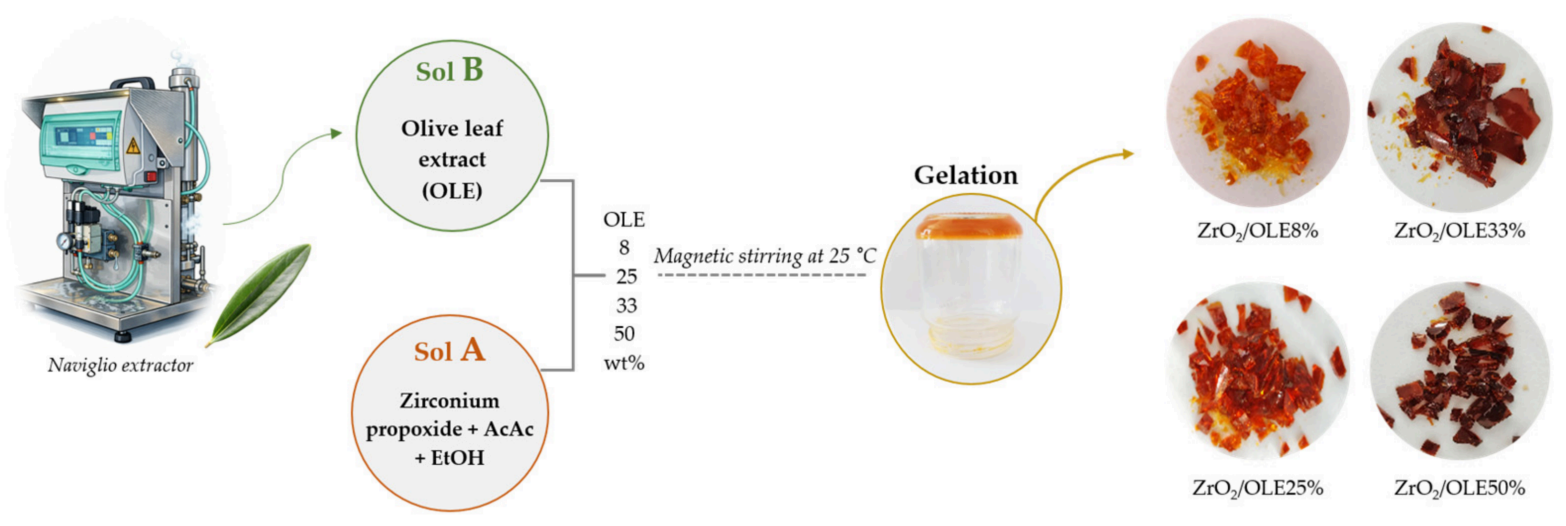
Flowchart of the Sol–gel synthesis of ZrO_2_/OLE hybrids.

ZrO_2_ and ZrO_2_/OLE hybrid materials containing 8, 25, 33, and 50 wt% of OLE were synthesized through the sol–gel route [26] following the scheme reported in Figure 10.

Briefly, zirconium (IV) propoxide 70 wt% in 1-propanol (Sigma-Aldrich Merck Life Science S.r.l., Milan, Italy) was used as the inorganic precursor, while ethanol (EtOH, ≥99.8% purity, Sigma-Aldrich Merck Life Science S.r.l., Milan, Italy) was employed as solvent and acetylacetone (AcAc, ReagentPlus, ≥99%, Sigma-Aldrich Merck Life Science S.r.l., Milan, Italy) as chelating agent to control the hydrolysis rate of the zirconium precursor. A first solution, named Sol A, was prepared by mixing zirconium (IV) propoxide with ethanol and acetylacetone, using the molar ratio EtOH:AcAc:Zr(OC_3_H_7_)_4_ = 6.5:0.3:1. The solution was stirred at room temperature until a homogeneous sol was obtained. Separately, olive leaf extract, previously obtained by Naviglio extraction, was dissolved in ethanol to prepare Sol B. Different amounts of OLE were used to obtain hybrid compositions containing 8, 25, 33, and 50 wt% of extract with respect to the final hybrid material. Sol B was then added dropwise to Sol A under magnetic stirring at room temperature to obtain sols that were left to undergo gelation. After gel formation, the wet gels were dried under vacuum until solid hybrid materials were obtained. The OLE loading was calculated with respect to the mass of ZrO_2_ in the final inorganic phase. Specifically, 0.08, 0.25, 0.33, and 0.50 g of OLE were added per 1.00 g of ZrO_2_, corresponding to theoretical OLE loadings of 8, 25, 33, and 50 wt% relative to ZrO_2_, respectively.

### 3.3. Colorimetric Analysis

For the colorimetric characterization, a PCE-CMS 6 colorimeter was used. The instrument operated using a D65 illuminant, corresponding to standard daylight conditions, produced by a blue LED light source. Measurements were performed with an 8/d observer geometry and a measurement aperture of 8 mm. The device allows measurements in the CIE Lab color space*. Prior to each analysis, the instrument was calibrated using a certified white reference standard to ensure measurement accuracy. Within the CIE Lab* system, L* represents the lightness parameter, ranging from black to white. The a* coordinate describes the green–red axis, where negative values indicate green tones and positive values correspond to red tones. Additionally, C* (chroma) expresses the color saturation or intensity, while h° (hue angle) defines the perceived color tone. The hue angle indicates whether a color tends toward red (0°), yellow (90°), green (180°), or blue (270°) [37]. Colorimetric measurements were carried out on five different samples: pure ZrO_2_ and ZrO_2_/OLE hybrids containing 8%, 25%, 33%, and 50% OLE. For each sample, three independent measurements were recorded on three different specimens to ensure reproducibility, and the standard deviation was calculated and reported as error bars. The color difference (ΔE) was calculated according to the following Equation (1) using pure zirconia (ZrO_2_) without OLE addition as the reference sample.

Δ*E* = √[(*L* − L*)^2^ + (*a* − a*)^2^ + (*b* − b*)^2^] (1)

### 3.4. FT-IR Analysis

FT-IR was employed to analyze the OLE and the hybrids. Spectra were collected using a Prestige21 Shimadzu system (Shimadzu, Milan, Italy) within the 4000–400 cm^−1^ range, with a resolution of 2 cm^−1^ and 64 scans per sample. For sample preparation, 2 mg of powdered substance was blended with 198 mg of dried KBr. The spectra were processed using IR solution software (version 1.60, Shimadzu, Milan, Italy), and graphs were generated using Origin (version 2024, OriginLab Corporation, Northampton, MA, USA).

### 3.5. Thermal Analysis

ZrO_2_ and ZrO_2_/OLE hybrids at different percentages of olive leaf extract were degraded in a Mettler TGA 1 Star System (Mettler-Toledo AG, Schwerzenbach, Switzerland). The thermobalance was calibrated by using the change in the magnetic properties of three metal samples (Isatherm, nickel alloy and Trafoperm 86) at their Curie points (148, 355 and 750 °C, respectively), following the procedure suggested by the manufacturer. Thermogravimetric (TG) runs were carried out in flowing nitrogen (0.06 L min^−1^) in the temperature range 25–800 °C by using a heating rate of 10 °C min^−1^. To correct the error in the mass determination due to the reduction in the buoyancy force at increasing temperature [38] a blank run, in the same experimental conditions, with an empty pan was carried out before the TG measurements and then subtracted from those containing, from time to time, about 15 × 10^–3^ g of hybrids. TG data for ZrO_2_ and related hybrids are reported as percentage of undegraded sample, (1 − *D*)% against temperature, where *D* = (Wo − W)/Wo, and Wo and W were the masses at the starting point and during scanning.

### 3.6. In Vitro Release

OLE release from the zirconia-based hybrids was evaluated in 95% (*v*/*v*) ethanol. Before the experiment, the materials were sieved to obtain a particle-size fraction of 75–125 μm. For each formulation, 50 mg of hybrid material was dispersed in 10 mL of release medium, corresponding to a material concentration of 5 mg mL^−1^. The suspensions were maintained at 25 °C under continuous magnetic stirring for 24 h. A calibration curve was prepared at 270 nm using pure OLE dissolved in 95% (*v*/*v*) ethanol at concentrations ranging from 0.003 to 1 mg mL^−1^. At each sampling time—5 min, 30 min, hourly from 1 to 7 h, and 24 h—a 1 mL aliquot was withdrawn and replaced with an equal volume of fresh 95% ethanol to maintain a constant total volume. Before UV–Vis analysis, the solid particles were separated from the collected aliquot using a sieve. The OLE concentration was determined using a T65 UV–Visible spectrophotometer equipped with an eight-cell motorized cell changer (PG Instruments, Leicestershire, UK). The cumulative release was expressed as a percentage of the theoretical amount of OLE initially present in the tested material:

(2) $$Cumulative\;OLE\;release\;\left(\%\right)=\;\frac{M_{t}}{M_{\mathrm{OLE},\mathrm{theoretical}}}\;\times \;100$$

where $M_{\mathrm{OLE},\mathrm{theoretical}}$ is the theoretical mass of OLE contained in 50 mg of each hybrid formulation, whereas $M_{t}$ is the cumulative mass of OLE released at time t. Error bars were calculated as the standard deviation of three independent measurements (*n* = 3). Ethanol at 95% (*v*/*v*) was selected as the release medium because concentrated ethanol is used, under specified conditions, as a substitute medium for food simulant D2 in migration studies concerning fatty-food contact materials [29].

### 3.7. Antibacterial Analysis

The antibacterial effectiveness of the evaluation was performed using a modified Kirby–Bauer method to assess the potential inhibitory effect of ZrO_2_/OLE hybrids on bacterial growth. For this purpose, the samples were sieved and compressed into 200 mg disks with a diameter of 1.3 cm, then UV-sterilized for 1 h before being placed in direct contact with microbial cultures. The synthesized materials were tested against two bacterial strains selected as bacterial species relevant for potential contamination of food products: *Escherichia coli* ATCC 25922 (Gram-negative) and *Enterococcus faecalis* ATCC 29212 (Gram-positive). *E. coli* was cultured on TBX Medium (Tryptone Bile X-Gluc, Liofilchem, Roseto degli Abruzzi, Italy) and incubated at 44 °C for 24 h. *E. faecalis* was cultured on Baird–Parker agar (Liofilchem, Italy) and incubated at 36 °C for 48 h. For both bacterial strains, plates inoculated with the bacteria under the same experimental conditions but without any material were used as negative controls. No inhibition zone was observed in the negative controls. After the incubation, the inhibition zones were measured, taking measurements for each Petri dish. The results were expressed as mean ± standard deviation (SD).

## 4. Conclusions

Olive leaf extract (OLE) was successfully incorporated into zirconia-based hybrids by the sol–gel method at theoretical loadings of 8, 25, 33, and 50 wt%. OLE also acted as a natural coloring component, shifting the yellow appearance of bare ZrO_2_ toward orange and orange–brown tones. The colorimetric response was not directly proportional to extract loading, with ZrO_2_/OLE25% showing the highest lightness and chroma, whereas the hybrids containing 33 and 50 wt% OLE were darker and less saturated. FT-IR analysis supported the incorporation of OLE-derived organic groups into the zirconia-based matrix. Changes in the O–H and Zr–O spectral regions indicated modifications of the local environment of the inorganic network, while the main zirconia-related bands remained detectable in all formulations. Thermal analysis showed a progressive shift in the main degradation stage toward higher temperatures with increasing OLE content, suggesting a stabilizing effect of the extract within the hybrid matrix. OLE loading also markedly affected the release behavior of the hybrids, with a transition from rapid release at low extract content to progressively stronger retention at higher loadings. The intermediate ZrO_2_/OLE25% formulation provided the most balanced behavior, combining an initially available fraction with a more gradually released fraction over time. These results indicate that the amount of OLE incorporated into the zirconia matrix can be used to modulate the release characteristics of the hybrid system. OLE incorporation did not produce detectable diffusion-mediated antibacterial activity against *Escherichia coli* or *Enterococcus faecalis* under the investigated conditions. Overall, ZrO_2_/OLE25% emerged as the formulation combining the most vivid coloration with the most suitable release behavior among the investigated compositions. These preliminary findings support further investigation into the valorization of olive leaf extract as a natural coloring and functional component in zirconia-based hybrid materials. Further studies should address the chemical composition and biological activity of the released fraction and evaluate the behavior of these materials under actual food-contact conditions.

## Figures and Tables

**Table 1:** DSC peak temperatures (*T*_p_), TG temperature at 5% mass loss (*T*_5%_), initial decomposition temperature (*T*_i_) (referred to the 2nd degradation stage), and solid residue at 800 °C for ZrO_2_ and ZrO_2_/OLE hybrids.

Sample	*T*_p_/°C	*T*_5%_/°C	*T*_i_/°C	*Residue*/%
**ZrO_2_**	115.1	105.1	296.2	74.4
**ZrO_2_/OLE8%**	118.0	102.9	300.9	72.8
**ZrO_2_/OLE25%**	120.3	99.6	306.1	70.3
**ZrO_2_/OLE33%**	123.7	93.2	318.2	70.2
**ZrO_2_/OLE50%**	126.4	91.1	320.4	69.3

## Data Availability

The original contributions presented in this study are included in the article. Further inquiries can be directed to the corresponding author.
